# Cerebral processing of sharp mechanical pain measured with arterial spin labeling

**DOI:** 10.1002/brb3.2442

**Published:** 2021-12-08

**Authors:** Vita Cardinale, Traute Demirakca, Tobias Gradinger, Markus Sack, Matthias Ruf, Nikolaus Kleindienst, Marius Schmitz, Christian Schmahl, Ulf Baumgärtner, Gabriele Ende

**Affiliations:** ^1^ Department of Neuroimaging, Central Institute of Mental Health, Medical Faculty Mannheim Heidelberg University Mannheim Germany; ^2^ Department of Neuroimaging and Core Facility ZIPP, Central Institute of Mental Health, Medical Faculty Mannheim Heidelberg University Mannheim Germany; ^3^ Institute of Psychiatric and Psychosomatic Psychotherapy, Central Institute of Mental Health Mannheim, Medical Faculty Mannheim Heidelberg University Mannheim Germany; ^4^ Department of General Psychiatry, Center for Psychosocial Medicine University of Heidelberg Heidelberg Germany; ^5^ Department of Psychosomatic Medicine and Psychotherapy, Central Institute of Mental Health Mannheim, Medical Faculty Mannheim Heidelberg University Mannheim Germany; ^6^ Department of Neurophysiology, Mannheim Center for Translational Neuroscience (MTCN), Medical Faculty Mannheim Heidelberg University Mannheim Germany; ^7^ Institute of Cognitive and Affective Neuroscience (ICAN) Medical School Hamburg Hamburg Germany; ^8^ Present address: Department of Biomedical Informatics of the Heinrich‐Lanz‐Center Medical Faculty Mannheim Heidelberg University Mannheim Germany

**Keywords:** acute pain, cerebral blood flow, functional neuroimaging, perfusion

## Abstract

**Introduction:**

Arterial spin labeling (ASL) is a functional neuroimaging technique that has been frequently used to investigate acute pain states. A major advantage of ASL as opposed to blood‐oxygen‐level‐dependent functional neuroimaging is its applicability for low‐frequency designs. As such, ASL represents an interesting option for studies in which repeating an experimental event would reduce its ecological validity. Whereas most ASL pain studies so far have used thermal stimuli, to our knowledge, no ASL study so far has investigated pain responses to sharp mechanical pain.

**Methods:**

As a proof of concept, we investigated whether ASL has the sensitivity to detect brain activation within core areas of the nociceptive network in healthy controls following a single stimulation block based on 96 s of mechanical painful stimulation using a blunt blade.

**Results:**

We found significant increases in perfusion across many regions of the nociceptive network such as primary and secondary somatosensory cortices, premotor cortex, posterior insula, inferior parietal cortex, parietal operculum, temporal gyrus, temporo‐occipital lobe, putamen, and the cerebellum. Contrary to our hypothesis, we did not find any significant increase within ACC, thalamus, or PFC. Moreover, we were able to detect a significant positive correlation between pain intensity ratings and pain‐induced perfusion increase in the posterior insula.

**Conclusion:**

We demonstrate that ASL is suited to investigate acute pain in a single event paradigm, although to detect activation within some regions of the nociceptive network, the sensitivity of our paradigm seemed to be limited. Regarding the posterior insula, our paradigm was sensitive enough to detect a correlation between pain intensity ratings and pain‐induced perfusion increase. Previous experimental pain studies have proposed that intensity coding in this region may be restricted to thermal stimulation. Our result demonstrates that the posterior insula encodes intensity information for mechanical stimuli as well.

## Graphical abstract

Using arterial spin labeling (ASL), one single block of sharp mechanical pain stimulation (96 s) induced perfusion increase in many areas of the nociceptive network. Moreover, a positive correlation between pain intensity ratings and perfusion increase in the posterior insula could be detected. Our results demonstrate that ASL constitutes a powerful alternative to BOLD fMRI for low‐frequency designs and that pain intensity coding in the posterior insula is not restricted to thermal stimulation.

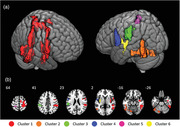

## 1 INTRODUCTION

Since its emergence, the field of non‐invasive functional magnetic resonance imaging (fMRI) has been dominated by studies using blood‐oxygenation level dependent (BOLD) imaging. BOLD fMRI is a powerful technique for functional paradigms, in which stimuli can be presented multiple times during an experiment. Arterial spin labeling (ASL) is an alternative fMRI method that is better suited to investigate single events, slow changes, or tonic processes such as the administration of a pharmacological agent, the induction of stress, mood states, or acute pain (Wang et al., 2003). As such, ASL is especially suited to investigate acute pain experimentally (Loggia et al., 2019).

Pain processing in patients diagnosed with borderline personality disorder (BPD) seems to be altered compared to healthy controls. Overall, pain sensitivity seems to be reduced in those patients (for a review see (Schmahl & Baumgärtner, 2015)). At the same time a substantial number of BPD patients show non‐suicidal self‐injurious behavior (NSSI) (Zanarini et al., 2008). Although there are several motives, one of the major reasons for NSSI is relief from aversive inner tension (Kleindienst et al., 2008). Most frequently used NSSI methods comprise tissue injury (Andover et al., 2010; Kleindienst et al., 2008; Turner et al., 2015). However, there is evidence that nociceptive stimulation rather than tissue injury contributes significantly to the reduction of aversive tension (Willis et al., 2017). Using BOLD fMRI to investigate pain processing in BPD may threaten the ecological validity of the experiment, as stimuli must be presented multiple times. As ASL can be used with low‐frequency designs, it constitutes an interesting alternative method.

To date, experimental pain studies using ASL in healthy controls utilized hot or cold temperature stimuli (Clewett et al., 2013; Frölich et al., 2012; Maleki et al., 2013; Owen et al., 2008; Zeidan et al., 2011; Zeidan et al., 2014), pressure (Frölich et al., 2012), deep muscular pain (Owen et al., 2010), or chemical noxious stimulation with capsaicin (Segerdahl et al., 2015). The most frequently applied NSSI method, however, is cutting (Kleindienst et al., 2008; Turner et al., 2015). To our knowledge, no ASL study so far has investigated pain responses to sharp mechanical pain. In order to address this gap in the literature, we employed a recently developed human surrogate model of incision pain (Shabes et al., 2016) to investigate the brain's response to sharp mechanical pain. The goal of the present study was to evaluate ASL as an application for examining neuronal pain processing in BPD patients. Specifically, we examined if ASL has the sensitivity to detect changes in perfusion within core areas of the nociceptive network applying a single block of sharp mechanical painful stimuli in healthy controls.

Three meta‐analyses of neuroimaging studies (Apkarian et al., 2005; Duerden & Albanese, 2013; Jensen et al., 2016) identified regions with high probability to be involved in pain processing in healthy subjects across pain types. According to their results, we hypothesized to find changes in perfusion due to painful stimulation in S1, S2, insula, ACC, PFC, thalamus, and cerebellum. Although the encoding of pain intensity is most probably the result of the dynamic interplay between many different brain regions of the nociceptive network (Wager et al., 2013), the posterior insula has been proposed to be a key node (e.g., Frot et al., 2007; Isnard et al., 2011; Segerdahl et al., 2015). It is involved in sensory processing of the pain experience (Bastuji et al., 2016; Frot et al., 2014), and a number of studies have found it to be associated with pain intensity coding (Bornhövd et al., 2002; Coghill et al., 1999; Frot et al., 2007; Iannetti et al., 2005). Moreover, it is the only cortical region where pain could be elicited by electrical stimulation (Mazzola et al., 2009). Using ASL, two studies found positive correlations between regional changes in blood flow within the insula and pain intensity ratings (Owen et al., 2010; Segerdahl et al., 2015). Since the combination of ASL with mechanical stimulation is novel, we wanted to be certain to focus on the area that would most likely show a response. Hence, as a proof of concept, we hypothesized that we could detect a positive correlation between perfusion in the posterior insula and pain intensity ratings using ASL and a single block of painful mechanical stimulation.

## 2 METHODS AND MATERIAL

### 2.1 Subjects

We analyzed datasets from 19 female healthy subjects (age range: 21–39, mean: 26.4 ± 6.2 standard deviation) that were included after screening to exclude any history of neurological and psychiatric conditions, chronic pain states, regular use of medication, and MRI contraindications. Informed consent was obtained, and experimental procedures were approved by the local ethics committee (2015‐600N‐MA) in accordance with the Declaration of Helsinki. We initially recruited 26 healthy subjects for this study, of which seven were excluded from the final analysis. Four subjects did not complete full screening, 1 dataset had to be dismissed due to technical problems during the MRI measurement, 1 due to strong subject movement, and another due to failure of the normalization procedure during preprocessing. All participants were recruited by advertisement in newspapers or flyers, distributed at the Medical Faculty Mannheim of the University of Heidelberg.

### 2.2 ASL technique

In ASL, arterial blood water is used as an endogenous tracer. This is accomplished by magnetically labeling spins in arterial blood water. There are different methods to achieve labeling (pulsed ASL, continuous ASL and pseudo‐continuous ASL), and each labeling scheme has its own strengths and weaknesses (see e.g., Borogovac & Asllani, 2012; Günther, 2014; for a comprehensive discussion and summary). It is important to note, however, that ASL has a lower SNR compared to BOLD fMRI. Specifically, only approximately 1% of the total signal is caused by blood delivered to the tissue. This disadvantage may be counterbalanced by decreased inter‐subject variability in ASL compared to BOLD fMRI (Liu & Brown, 2007). However, ASL techniques have constantly been improved over the years with respect to SNR. For an overview of recent advancements in ASL, please see Hernandez‐Garcia et al., 2019. According to the recommendations of the ISMRM Perfusion Study Group and the European Consortium for ASL in Dementia (Alsop et al., 2014), we employed a sequence with the pseudo‐continuous labeling technique, in which arterial blood water is magnetically labeled in a plane beneath the brain and perpendicular to the main feeding arteries in the neck. The labeled blood is given time to diffuse into the capillary system of the brain before the MR image is read out. In order to obtain a perfusion‐weighted image, a second image is needed in which no labeling is applied. Because the magnetic label reduces the signal in the image, the labeled image needs to be subtracted from the non‐labeled image in order to obtain a perfusion‐weighted image. As a consequence, any slow drifts present in the signal are removed and data points in a time‐series of images can be meaningfully compared to each other. Moreover, perfusion values can be quantified in absolute units of ml/g/min.

### 2.3 Mechanical pain stimulation device

**FIGURE 1:**
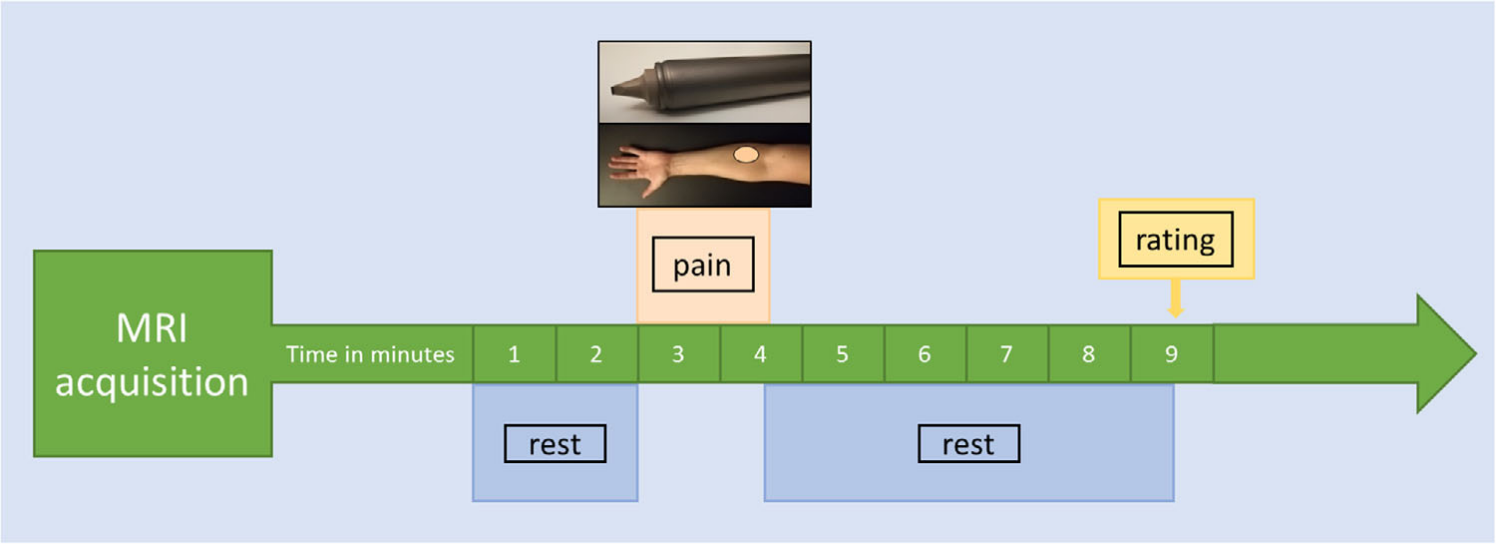
Schematic representation of the paradigm. Mechanical pain was applied with a blunt blade to the left forearm within an area of approximately 7 cm^2^ (shaded area in light red) for 96 s. Pain ratings were acquired 5 min after pain offset

Shabes et al. introduced a stimulation device as a model of sharp mechanical pain. The device consists of a blunt blade of 4 mm length and 100 μm width that is attached to a plastic mounting and a steel tube (see Figure 1; see also Shabes et al., 2016). A moving weight (4096 mN) inside the tube ensures that the blade can be applied with constant force. In their study, Shabes et al. showed that the pain experience during 7 s of stimulation with a blunt blade is comparable to the pain experience after an incision with respect to sensory and affective properties (Shabes et al., 2016). The stimulator was developed according to the findings of Greenspan and McGillis (1991) and Slugg et al. (2004, 2000) who tested pin‐prick stimuli of different force and tip dimensions in psychophysical experiments in humans and single fiber recordings in monkeys (Greenspan & McGillis, 1991). These stimuli primarily activated *Aδ*‐nociceptors together with C‐fiber nociceptors. Since the stimulator touches the skin, *Aβ*‐fibers are likely to be coactivated as well, but with much less specificity and brief transient firing (Perl, 1968).

### 2.4 Duration of stimulation block

ASL is well suited for low‐frequency paradigms (Aguirre et al., 2002; Wang et al., 2003), in which few but relatively long experimental events are under investigation, such as the effect of pharmacological agents or psychological and physiological states. An example for the latter category is acute pain. Most experimental pain studies using ASL applied stimulation that added up to at least 2 min of total stimulation time (2 min: Maleki et al., 2013; 3 min: Zeidan et al., 2011; 5 min: Frölich et al., 2012; Owen et al., 2008). Others have employed paradigms with more than 15 min of painful stimulation (Owen et al., 2010; Segerdahl et al., 2015). Aguirre et al. have shown that at task frequencies below 0.009 Hz perfusion MRI starts to outperform BOLD fMRI in terms of relative sensitivity (Aguirre et al., 2002). That corresponds to a blocked design wherein a 60 s task alternates with 60 s of baseline measurement. Wang et al. demonstrated that the functional signal‐to‐noise‐ratio (SNR) on group maps, based on perfusion measurements, are relatively constant between designs with blocks of 30, 60, 150, and 300 s of finger tapping, respectively (Wang et al., 2003). In order to increase ecological validity, we applied only one block of stimulation with no repetition. We tested 64 versus 96 s of painful stimulation in a pre‐pilot study (unpublished data) and decided for the longer stimulation duration of 96 s for the present study, as it confers higher sensitivity to detect pain‐related changes.

### 2.5 Assessment of mechanical pain thresholds and pain sensitivity

#### 2.5.1 Pain thresholds

Mechanical pain thresholds were quantified using seven different forces of pin‐prick stimuli: 8, 16, 32, 64, 128, 256, and 512 mN. These stimuli are part of the quantitative sensory testing protocol by the German Research Network on Neuropathic Pain (Rolke et al., 2006). Pin‐pricks were applied at a rate of 2 s “on”/2 s “off” in ascending order until participants reported a sharp sensation and in descending order until participants reported a blunt sensation. This procedure was repeated five times for each subject, and the mean of the five thresholds was used as a final measure of the average mechanical pain threshold.

#### 2.5.2 Pain intensity and unpleasantness

Ratings of acute blade‐induced pain were acquired after the scanning sessions, to prevent confounding changes in perfusion rates due to rating and motor response activity. Participants had to verbally indicate on a numerical rating scale ranging from 0 to 100 how intensely (0 = “no pain at all”, 100 = “most intense pain imaginable”) and aversively (0 = “no pain at all”, 100 = “most aversive pain imaginable”) they perceived the stimulation. That is, we acquired a single pain intensity rating and a single pain unpleasantness rating for the complete pain stimulation period (96 s) 5 min after stimulation offset.

### 2.6 Experimental design

The MRI measurement started with a baseline scanning period of 120 s to ensure that the subjects had the opportunity to get used to the scanner and measurement noise and the ASL signal could stabilize. The baseline measurement was followed by 12 consecutive pain stimuli that were applied to the left forearm within an area of approximately 7 cm^2^. The blade stimulator was hand‐held and slightly moved from one application to the next in order not to stimulate the same spot repeatedly. The application did not follow a specific pattern. The blade orientation varied randomly. Each single stimulus lasted 6 s followed by 2 s stimulation offset, resulting in one stimulation block lasting 96 s in total. In order to ensure that the stimulus timings were exact, we programmed a visual display that was projected onto a monitor behind the scanner. The monitor was only visible to the experimenter but not to the subject. For the entire stimulation period, with the start of each measurement (i.e., 12 times 8 s), the display counted down from 6 to 1 in 1‐s increments followed by the display of the word “break” for 2 s during which the blade stimulator was moved to the next stimulation site. The timing of stimulation offsets was chosen to fall within the labeling interval of each image acquisition to guarantee acute pain during image read‐out. After pain stimulation, the scanning was continued for 5 min. Pain ratings were collected at the end of the measurement. We did not collect pain ratings immediately following the pain stimulation because our study was a proof‐of‐concept study designed to prepare for a study in which a “pain only”‐condition is going to be compared to a condition in which pain will be applied after the induction of stress in a Borderline patient cohort. In the latter condition, our interest will be to observe the evolution of the stress response over approximately 5 min. In order not to contaminate the stress response in the latter condition with a systematic motor and evaluation response, it will be necessary to collect the pain rating after the stress has evolved. Hence, we decided to apply a similar delay between pain application and collection of pain ratings in our present study. See Figure 1 for schematic representation of our proof‐of‐concept experiment.

### 2.7 MRI acquisition

All imaging data were collected using a 3‐T Siemens Trio scanner with a 32‐channel head coil (Siemens, Healthineers, Erlangen, Germany). We acquired a high‐resolution, T1‐weighted structural image from each subject using a three‐dimensional magnetization prepared rapid acquisition gradient echo (MPRAGE) pulse sequence (TR/TE/TI = 2300/3.03/900 ms; flip angle, 9°; 192 sagittal slices; voxel size, 1.0 × 1.0 × 1.0 mm^3^). Functional images were acquired using a pCASL perfusion imaging sequence using the 3D GRASE read‐out technique (Fernandez‐Seara et al., 2005; Günther et al., 2005). A bolus length of 1800 ms and a post labeling delay of 1500 ms in duration were applied using a fixed labeling plane offset of 90 mm. Other parameters were: FoV readout 220 × 220 mm, FoV phase 75%, 64 × 64 matrix, 5/8 partial Fourier, pre‐scan normalization, 24 slices, slice thickness and gap each 5 mm, phase encode direction R > > L, background suppression, TR = 4000 ms, and TE = 32.2 ms. We oriented the read‐out volume parallel to the ACPC‐plane.

### 2.8 Image analysis

We used FSL (https://fsl.fmrib.ox.ac.uk/fsl/) analysis tools (version 5.0.9) from the Oxford Centre for Functional Magnetic Resonance Imaging of the Brain (fMRIB, Oxford, UK) to preprocess our data. First, we extracted brain tissue with the anatomical images using the brain extraction tool “BET”(Smith, 2002). We then corrected the functional images for motion using “MCFLIRT”, a motion correction tool based on optimization and registration techniques used in FLIRT, a fully automated tool for linear intra‐ and inter‐modal brain image registration (Jenkinson et al., 2002). We collected our data with two phase‐encode directions, resulting in pairs of images with distortions going in opposite directions. From these pairs, the susceptibility‐induced off‐resonance field was estimated using a method similar to that described in Andersson et al., 2003 as implemented in FSL (Smith et al., 2004), and the two images were combined into a single corrected one as preparation for “TOPUP”. Madai et al. (2016) showed that applying top‐up to perfusion data acquired with 3D‐GRASE readout helps to improve data quality (Madai et al., 2016). After correcting the functional time series for susceptibility induced distortions using “TOPUP” and “APPLYTOPUP”, we registered the motion‐corrected functional images to the brain‐extracted anatomical image using “EPI_REG”, a script designed to register functional or diffusion images to structural images (Jenkinson & Smith, 2001; Jenkinson et al., 2002). We then brain‐extracted the functional images by applying the binary brain mask of the skull‐stripped anatomical image to the functional image time‐series.

### 2.9 Statistical analysis

#### 2.9.1 Pain activation

We used the fMRI analysis tool “FEAT” to analyze the single‐subject data (Woolrich et al., 2001). We performed analyses on the time series of difference images of each subject and used a block design to model pain activation against baseline activation. In “FEAT”, we modeled one single explanatory variable according to the timing of the whole pain stimulation block and convolved the resulting regressor using a standard hemodynamic response function. Specifically, we used a single regressor for the complete pain stimulation period of 96 s and not a regressor for each single pain stimulus. Group activation maps were generated by fMRIB local analysis of mixed effects (FLAME1) tool (Woolrich et al., 2004). To correct for multiple testing, a cluster correction method based on Gaussian random field theory was applied (Friston et al., 1991; Nichols, 2012; Worsley et al., 1992).

One problem that can occur with unsegmented 3D GRASE read‐out, as applied in the present study, is a blurring artifact in encoding direction that can hamper the spatial specificity of identified perfusion changes (Liang et al., 2014). As a consequence, the probability of false positive results is enhanced, since the signal of voxels with high *z*‐values may influence neighboring slices. To correct for this enhanced probability for false positives and to enhance spatial specificity, group activation maps were thresholded at *z* > 3.5, following the recommendations of Woo et al. (Woo et al., 2014). The clusters were controlled at a family wise error rate of .05.

#### 2.9.2 Region of interest analysis within the posterior insula: Correlation between perfusion and pain thresholds, intensity, and unpleasantness ratings

**FIGURE 2:**
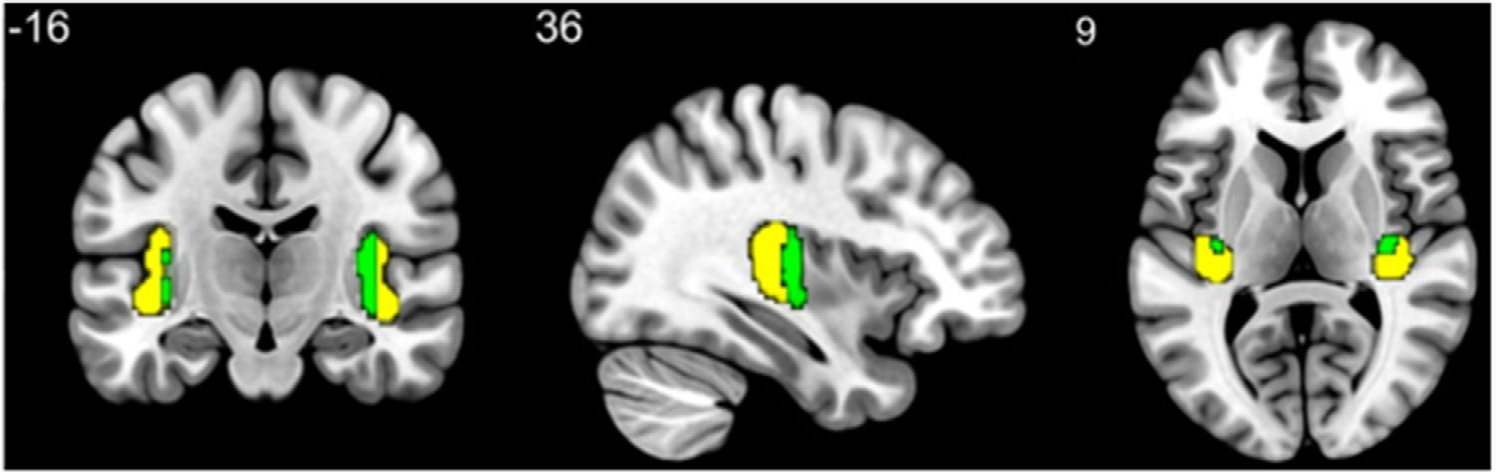
Brain mask used in the ROI analysis. The yellow‐colored area represents the posterior insula as defined in the Juelich histological atlas, thresholded at a probability value of 0.2 and binarized. The area shaded in green represents the statistically significant voxels of the pain activation that intersected with the brain mask. The ROI analysis was performed with the average perfusion values of the voxel within the green area

In a region of interest (ROI) analysis, we correlated pain intensity and unpleasantness ratings, as well as pain thresholds with perfusion values within the posterior insula, to test the specific hypothesis that pain intensity is positively correlated with perfusion in the posterior insula. Per subject, we averaged the perfusion values over 96 seconds stimulation time within voxels that fell into the intersection of our corrected statistical image, and the posterior insula mask according to the Juelich histological atlas in FSL (Eickhoff et al., 2006; Eickhoff et al., 2007). In Figure 2, the posterior insula as extracted from the histological atlas is depicted in yellow and green, where the green area depicts the area of overlap between the atlas mask and statistical mask (i.e., the binarized cluster‐corrected activation mask). Correlations were calculated across voxels within the green area. We must assume limited power of our paradigm because of inherently low SNR of ASL, relatively short stimulation protocol, and small sample size. In order to minimize the probability of a Type II error, we decided to conduct our analysis in this subregion. After spatially averaging, we normalized the average perfusion during pain stimulation to the average baseline perfusion. The distribution of all variables of interest (normalized perfusion, pain intensity, pain unpleasantness, and mechanical thresholds) was examined with respect to normality using Shapiro–Wilk tests. Mechanical pain thresholds were normally distributed after logarithmical transformation. Hence, for following analyses, we used logarithmically transformed thresholds. For an overview of descriptive information of pain thresholds, intensity, and unpleasantness ratings, see Table 2 and Figure 6. Finally, we calculated correlations between normalized perfusion during pain and pain intensity ratings, as well as pain mechanical pain thresholds using nonparametric estimates (Spearman's rho) in SPSS (version 22.0). We decided to use a nonparametric instead of parametric estimation of correlation for two reasons: First, pain rating scales should be treated as ordinal rather than interval scale variables because ratings are highly subjective and these rating scales may not have reliable ratio properties, resulting in the possibility that the distances between the rating‐values may not be equal. Second, it is unclear whether the relationship between perfusion and pain ratings is of a linear nature or not. As we hypothesized a positive relationship between pain ratings and perfusion, we performed one‐sided tests of significance.

## 3 RESULTS

### 3.1 Pain activation after whole‐brain cluster‐correction

**FIGURE 3:**
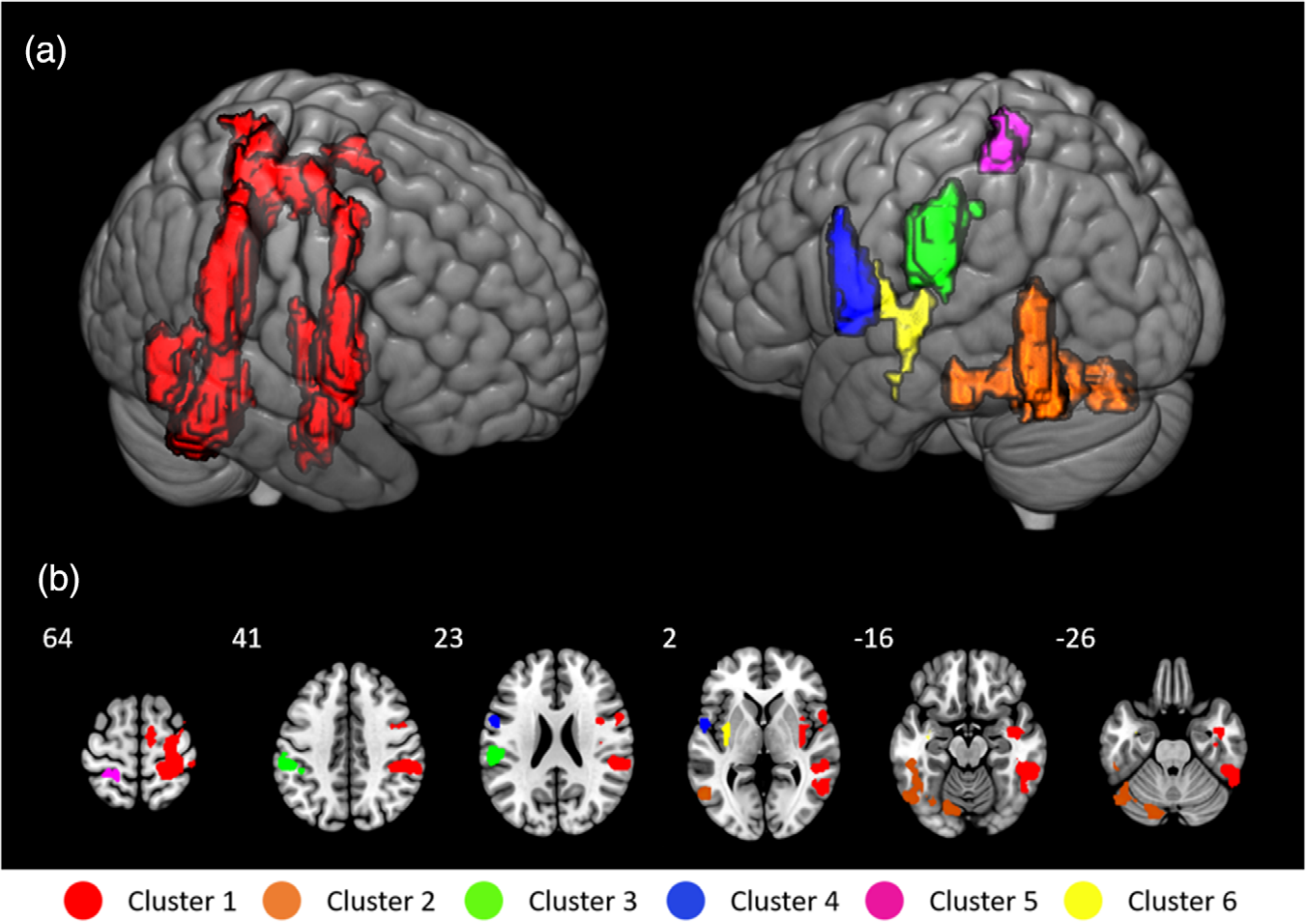
(a) 3D view of significant clusters after cluster correction (cluster‐building threshold z > 3.5, p = .05). (b) Transversal view of the clusters at Z = 64/41/23/2/−16/−26, respectively

We found increased perfusion in response to painful stimulation, but no decreases. Six clusters reached significance after running a whole‐brain analysis with a cluster building threshold of *z* = 3.5 and a significance threshold of *p* = .05 (Figure 3). The largest cluster (red) was located contralateral to the stimulation site within the right hemisphere and expanded from the precentral (supplementary motor area and premotor cortex) and postcentral gyrus (primary and secondary sensorimotor cortex) to the insula and inferior parietal lobe. Local maxima of the cluster were located within the inferior parietal lobe, S1, S2, parietal operculum, and insula (see Table 1). Perfusion significantly increased in five other clusters within the left hemisphere. The largest of the five ipsilateral clusters was located within the inferior and middle temporal gyrus, temporo‐occipital lobe, and cerebellum (orange). The other clusters were located within the inferior parietal lobe and parietal operculum (green), S2 and premotor cortex/BA6 (blue), superior parietal lobe and S1 (pink) and posterior insula, anterior insula, putamen, as well as within white matter near the laterobasal group of the amygdala (yellow). An overview of the local maxima of all clusters is provided in Table 1.

**TABLE 1:** Peak voxels within clusters of significant pain‐related perfusion increase. Cluster‐building threshold *z* > 3.5, cluster significance threshold *p* = .05

		Anatomical label		MNI coordinates				
Cluster Nr.	Cluster size (Nr. of voxels)	Talairach Deamon label	AAL	X	Y	Z	*z*‐Value	*p*‐Value
1	6541							1.23e^−13^
		Postcentral gyrus	SupraMarginal_R	52	−32	38	5.21	
		Postcentral gyrus	Postcentral_R	28	−38	58	5.2	
		Insula	NA	38	−4	−6	5.16	
		Postcentral gyrus	Postcentral_R	20	−38	68	5.07	
		Inferior parietal lobule	SupraMarginal_R	58	−34	28	4.9	
		Precentral gyrus	Rolandic_Oper_R	58	4	12	4.85	
2	1667							4.89e^−06^
		Middle temporal gyrus	Temporal_Mid_L	−56	−64	6	4.57	
		Declive	Cerebelum_Crus1_L	−48	−68	−26	4.08	
		Middle temporal gyrus	Temporal_Inf_L	−52	−34	−18	4.08	
		Declive	Cerebelum_Crus1_L	−14	−78	−22	4.05	
		Declive	Cerebelum_6_L	−18	−76	−22	4.05	
		Superior temporal gyrus	Temporal_Mid_L	−42	−52	−20	4.01	
3	863							.00032
		Inferior parietal lobule	SupraMarginal_L	−56	−32	32	4.73	
		Postcentral gyrus	SupraMarginal_L	−50	−24	24	4.4	
		Inferior parietal lobule	SupraMarginal_L	−58	−26	24	4.22	
		Inferior parietal lobule	SupraMarginal_L	−56	−30	24	4.21	
		Insula	Temporal_Sup_L	−56	−32	18	4.14	
		Inferior parietal lobule	Parietal_Inf_L	−42	−38	40	3.67	
4	508							.00305
		Precentral gyrus	Rolandic_Oper_L	−56	2	8	5.23	
		Precentral gyrus	Precentral_L	−58	4	32	4.18	
5	259							.0208
		Sub‐gyral	NA	−20	−44	60	4.3	
		Inferior parietal lobule	Postcentral_L	−28	−42	58	4.23	
		NA	NA	−32	−42	70	3.67	
6	237							.0252
		Claustrum	NA	−36	−10	−2	4.51	
		Extra‐nuclear	NA	−34	−4	2	4.46	
		Insula	Insula_L	−36	−2	12	4.17	
		Claustrum	Insula_L	−34	−16	8	4.1	
		Sub‐gyral	NA	−36	−6	−20	3.9	

To summarize, after cluster‐correction on the statistical map, we found significant increases in perfusion due to painful stimulation in multiple areas across the cortex. We found significant bilateral increases in S1, S2, premotor cortex, insula, inferior parietal lobe, and parietal operculum. Voxels in the supplementary motor area were only significantly increased contralateral to the stimulation site. Overall, the contralateral cluster contained more voxels compared to the ipsilateral clusters, especially in precentral regions.

### 3.2 ROI analysis of the posterior insula

**FIGURE 4:**
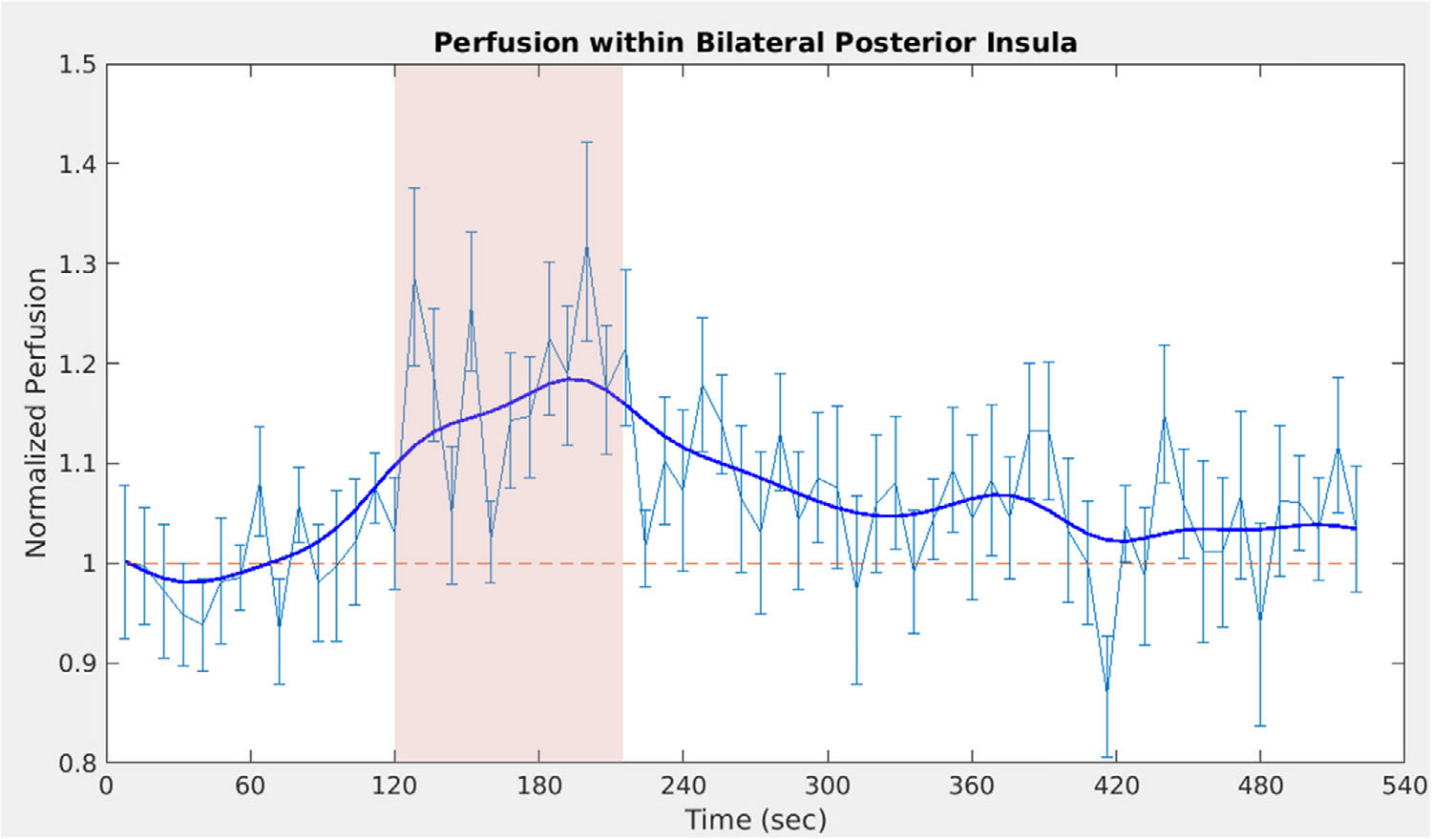
Normalized perfusion within the posterior insula (mask see Figure 3). Red dashed area: Time window in which noxious stimuli were applied. Red stippled line: Baseline perfusion in the absence of pain. Blue line: Perfusion averaged across 19 subjects with error bars (± standard error of the mean). Solid blue line: Average perfusion smoothed for better visualization

**FIGURE 5:**
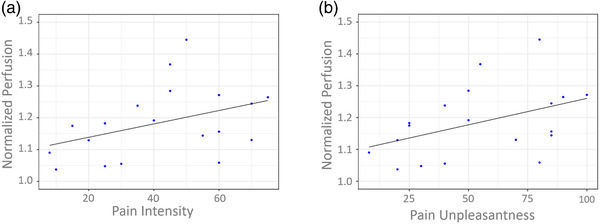
Correlation (Spearman's rho) between normalized perfusion within our posterior insula mask and (a) pain intensity: medium correlation (*r* = .444, *p* = 0.028) and (b) pain unpleasantness: large correlation (*r* = .506, *p* = .014). For the purpose of visualization, regression lines are plotted

Figure 4 shows the temporal evolution of the perfusion signal within the intersection of cluster 1 (see Figure 3 and Table 1) and the posterior insula (Figure 2, area shaded in green), averaged across all subjects. Normalized perfusion values were calculated per subject by averaging across the full stimulation period of 96 seconds (i.e., 12 TRs). We expected to find a positive correlation between intensity rating and perfusion change within the posterior insula. In SPSS, we calculated nonparametric correlation estimates and performed a one‐sided test of significance, as we hypothesized a positive correlation. First, pain intensity and pain unpleasantness ratings were highly correlated (rho = 0.94, *p* < .001). Both ratings showed a positive correlation with normalized perfusion values within the posterior insula (intensity: rho = 0.44, *p* = .028; unpleasantness: rho = 0.51, *p* = .014; see Figure 5). However, pain thresholds were not correlated with either intensity, unpleasantness ratings or normalized perfusion during pain (intensity: rho = −0.03, *p* = .46; unpleasantness: rho = 0.03, *p* = .45; normalized perfusion: rho = −0.14, *p* = .28; one‐tailed tests).

### 3.3 Psychophysical data

**FIGURE 6:**
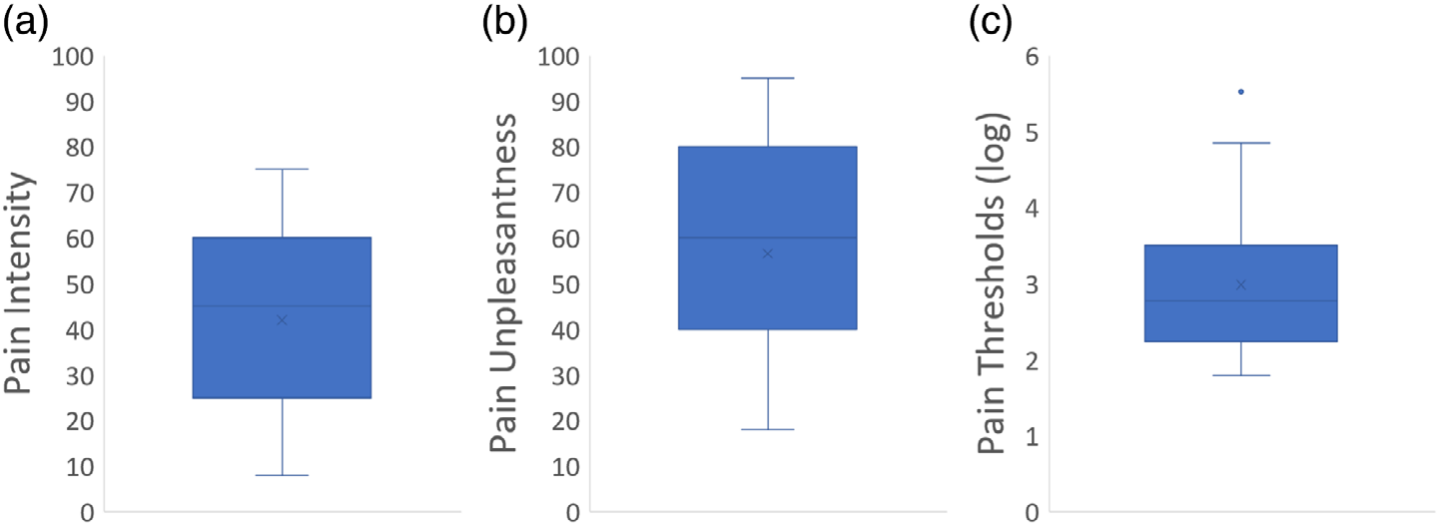
Boxplot diagrams of (a) pain intensity ratings, (b) pain unpleasantness ratings, and (c) log‐transformed mechanical pain thresholds. Middle line = median; x = mean value; lower and upper bound = end of first and third quartile; whisker = minimum and maximum value; blue dot = outlier (defined as 1.5 times the interquartile range)

On average, our subjects experienced moderately intense and unpleasant pain. However, variance within the parameter was relatively high (pain intensity mean: 42.0 ± 21.1, standard error of the mean (SEM) = 4.9; pain unpleasantness mean: 54.6 ± 28.9, SEM = 6.6, see Table 2). This may be related to the fact that we used a pain stimulator with a fixed force. Thus, we did not match the strength of nociceptive stimulation to the individual mechanical pain thresholds (Figure 6).

**TABLE 2:** Pain intensity rating scale: 0–100; 0 = “no pain at all“, 100 = “most intense pain imaginable". Pain unpleasantness scale: 0–100; 0 = “not aversive at all”, 100 = “maximally aversive”. Mechanical pain threshold has been logarithmized due to non‐normal distribution. Range of raw values: 4–512 (mN)

				Mean		Std. Deviation
	N	Minimum	Maximum	Statistic	Std. Error	Statistic
Pain intensity	19	8.00	75.00	42.00	4.85	21.15
Pain unpleasantness	19	8.00	100.00	54.63	6.62	28.87
Mechanical pain threshold (ln)	19	1.79	5.52	2.99	.23	1.00

## 4 DISCUSSION

In summary, we found that one block of 96 seconds with sharp mechanical painful stimulation was related to an increase in perfusion in several regions across the cortex including S1, S2, posterior insula, operculum, parietal and temporal gyrus, premotor cortex, temporo‐occipital lobe, and the cerebellum. We did not detect significant increases in perfusion within the ACC, thalamus, or PFC as hypothesized and nor did we detect perfusion decreases in response to sharp mechanical pain. However, we found a significant correlation between pain intensity and unpleasantness ratings, and posterior insula perfusion in an ROI analysis.

Within the nociceptive network, the posterior insula is involved in sensory processing of the pain experience (Bastuji et al., 2016; Frot et al., 2014) and pain intensity encoding (Bornhövd et al., 2002; Coghill et al., 1999; Frot et al., 2007; Iannetti et al., 2005). In line with these findings, we found a positive correlation between perfusion changes due to noxious mechanical stimulation in the posterior insula and pain intensity and unpleasantness ratings. However, the insula is a functionally heterogeneous region also involved in sensorimotor and affective processing, as well as higher‐level cognition (Kurth et al., 2010; Uddin et al., 2017). Structurally, the central insular sulcus separates the anterior from the posterior portion. Both the posterior and anterior insula have been shown to play a role in pain processing. Whereas the anterior insula is rather associated with the emotional component of pain, the posterior insula is involved in processing of sensory aspects of pain (Bastuji et al., 2016; Craig, 2002; Frot et al., 2014).

Although pain intensity coding is not exclusively related to the posterior insula, many studies conclude that the posterior insula may constitute a key region for pain processing (Isnard et al., 2011; Mazzola et al., 2009; Segerdahl et al., 2015). Two studies have demonstrated the relationship between posterior insula and pain perception by observing behavioral responses following experimental insular stimulation or pathologic excitation. Mazzola et al. showed that electrical stimulation of the posterior insula elicited pain sensations in 27% of a larger sample of epilepsy patients (Mazzola et al., 2009). In another study investigating epilepsy patients, spontaneous painful seizures originated from the posterior third of the insula, as measured with intracerebral field potentials (Isnard et al., 2011), highlighting the role of the posterior insula in pain processing. Using ASL, Owen et al. investigated muscular pain following a hypertonic saline injection and found a strong correlation between perfusion across the entire insula and pain intensity ratings (Owen et al., 2010). Segerdahl et al. used ASL to investigate capsaicin‐induced pain and found that perceived pain intensity correlated positively only with perfusion in the posterior insula but no other brain region in a whole brain analysis (Segerdahl et al., 2015). Our finding is in line with these studies.

There is an ongoing discussion whether the response of the posterior insula to nociceptive stimulation is actually pain‐specific (e.g., Garcia‐Larrea & Peyron, 2013) or rather, salience‐related (e.g., Iannetti & Mouraux, 2010; Legrain et al., 2011; Mouraux et al., 2011). On the other hand, Wager et al. (2013) identified a neurologic signature of noxious heat pain using fMRI (Wager et al., 2013). Applying machine learning analysis, they demonstrated that a pattern of fMRI activity across S2, ACC, thalamus and periaqueductal gray, and anterior and posterior insulae, among others, predicted individual pain intensity. The work by Andrew and Craig (2002) suggests input from nociceptive spinal neurons in lamina I to the thalamus and dorsal posterior insula (Andrew & Craig, 2002). Several authors have suggested that the posterior insula may respond to painful thermal stimulation because it is preferentially involved in thermoception (Craig et al., 2000; Hua et al., 2005; Liberati et al., 2018). Craig and colleagues have shown that the posterior insula is involved in both thermoreception and the experience of pain even without nociceptive input during the thermal grill illusion (Craig, 2004). Based on these and subsequent findings, Craig suggested a compelling concept, after which the dorsal insula served as a sensory input region for thermal and nociceptive stimuli, whereas the anterior part of the insula could be a homeostatic control center with its dense connections to the autonomic and limbic‐emotional systems (Craig, 2002, 2003). Our result is in line with this concept since it indicates that the posterior insula encodes pain intensity for non‐thermal stimuli as well. Hence, despite the question as to whether the posterior insula represents a nociception‐specific or rather salience‐related hub, our data suggests that this region is not only involved in intensity coding for thermal pain, but also mechanical pain. We clearly cannot dissociate effects of pain and salience with our paradigm. However, to our knowledge, this is the first neuroimaging study to find a positive correlation between sharp mechanical pain intensity ratings and posterior insula perfusion. As such, the positive correlation between perfusion and pain intensity ratings within the posterior insula adds a new piece of evidence to the discussion. Moreover, our finding demonstrates the power of ASL as functional neuroimaging method considering the relatively short stimulation time of our pain paradigm.

Many of the regions that were activated in our study are regions typically found across pain studies and represent different components of the pain experience such as sensory‐discriminative, motor, affective, and cognitive aspects. Bilateral activation of S1 and S2 as sensory‐discriminative responses, despite unilateral stimulation, is an established phenomenon (Duerden & Albanese, 2013; Peyron et al., 2000).

It is well known for somatosensory non‐noxious stimuli that their cortical representations are contralateral for S1 responses and bilateral for responses in S2 with a larger and earlier response in the contralateral than in the ipsilateral hemisphere relative to the side of stimulus application (e.g., Jung et al., 2009). For nociceptive stimuli, similar findings were reported, showing contralateral responses within S1 and bilateral in the operculoinsular cortex without significant delay between both hemispheres. This may hint at direct bilateral targeting from the thalamus rather than at interhemispheric connections from the contralateral to ipsilateral hemisphere (Schlereth et al., 2003). In functional MRI, temporal resolution is too coarse to test hypotheses about differences in activation timing or latency. Additionally, statistical thresholds must be applied to deal with the problem of multiple comparisons. Activities in the operculum and the insula are typically found bilaterally (Apkarian et al., 2005; Garcia‐Larrea et al., 2003), but can be detected in the hemisphere contralateral to the stimulation site already at higher thresholds. Lowering the statistical threshold (Baumgärtner et al., 2010; Mouraux et al., 2011) or increasing the gain of stimulation, for example, by application of capsaicin (Maihöfner & Handwerker, 2005), typically reveals a bilateral response pattern. Both laser heat stimuli and mechanical pin‐prick stimuli have been demonstrated to result in bilateral activation of both posterior and anterior insula in the same study. However, a hand–foot somatotopy was only found in the contralateral insula (Baumgärtner et al., 2010). Since we applied relatively strict statistical thresholds, our result of bilateral posterior insula activation may reflect the fact that our subjects perceived the stimulation as painful rather than non‐painful.

Regarding the activation of motor‐related areas, the exact location of pain‐related activation could shed further light on the nature of the stimulus. If the representation were mainly in area 3b, it would coincide with representations of tactile superficial stimuli. So far, nociceptive stimuli activating S1 have been demonstrated to be represented either in area 3a, 1, or even further posterior (Ploner et al., 2000; Schlereth et al., 2003; Whitsel et al., 2009). Unfortunately, given the spatial resolution in our study, we are unable to make a distinction between the different sub‐areas. The activity cluster covering S1 included the whole postcentral gyrus, parts posterior to it, and parts of the precentral gyrus (M1). Moreover, activation of motor‐related areas such as the SMA and premotor cortex may be related to the suppression of movement (Apkarian et al., 2005; Owen et al., 2010). Also, the cerebellum frequently is activated in pain studies (Apkarian et al., 2005; Jensen et al., 2016). Although its role in pain processing is not yet fully understood, the cerebellum may contribute to the integration of sensorimotor, affective and cognitive processes necessary to produce the multidimensional pain experience (Moulton et al., 2010).

Although having a high likelihood to be activated by pain, we did not find any changes in ACC, PFC, or thalamus (Apkarian et al., 2005; Duerden & Albanese, 2013; Jensen et al., 2016). This may be a consequence of the specificity of sharp mechanical pain compared to other types of pain, and/or a lack of power due to the experimental set‐up and MR data quality.

Maihöfner and Handwerker (2005) conducted the only study to our knowledge that directly compared sharp mechanical pain to heat pain using BOLD fMRI (Maihöfner & Handwerker, 2005). They found that thermal hyperalgesia resulted in higher activation of inferior, middle and superior frontal cortex, as well as anterior insula and cingulate cortex when compared to hyperalgesia induced by pin‐pricks, despite no difference in intensity ratings. That is, mechanical pain may elicit lower perfusion increases in frontal and cingulate cortices compared to heat induced pain.

A reason for this difference in activation pattern may be associated with the type of receptors that are excited by mechanical compared to thermal stimulation. Our blade stimulator primarily excites nociceptive Aδ‐fibers with minor contribution of C‐fibers (Slugg et al., 2004, 2000). Aβ‐fibers are also likely to be activated, however to a lesser degree, since these fibers and related receptors are mostly sensitive to velocity and not as much to force (Perl, 1968). Given these findings, contribution of Aβ‐fibers for our outcome should be minor but cannot be excluded.

Other studies have found that pin‐pricks, that is mechanical stimuli with a relatively small contact area, excite the lateral pain system (S1, S2, dorsal insula) more frequently than the medial pain system (anterior or mid‐cingulate gyrus) (Seifert et al., 2008). In our results, stimulation related activation occurred within the posterior rather than the anterior insula. As such, this may reflect specificity of mechanical stimulation, but without direct comparison to other types of stimulation of same intensity, this interpretation remains speculative. Ultimately, the question to what extent our results may represent a specific response to sharp mechanical pain needs to be clarified in future studies.

Apart from the possibility that our response pattern reflects specificity of mechanical pain, it is possible that we failed to find significant changes within the cingulate and prefrontal cortex, as well as the thalamus, because we did not match the painfulness of the stimulus with the individual mechanical pain thresholds, that is, there was a high variance with respect to pain intensity and unpleasantness ratings among our sample. Kong et al. conducted an experimental pain study using BOLD fMRI in 61 healthy subjects and were able to show that less painful stimuli elicited significantly different activation patterns compared to highly painful stimuli (Kong et al., 2010). They demonstrated that stimuli perceived as less painful elicited deactivations in a larger network compared to highly painful stimuli. Hence, in our sample heterogeneity in the brain response due to large variance in the subjective pain intensity levels might have prevented changes in perfusion to reach significance on group level.

Another important aspect influencing the power of an MR study is the data quality, which heavily depends on the specific sequence set‐up. We decided to use a pCASL 3D GRASE with unsegmented read‐out for the sake of higher temporal resolution. However, the single‐shot read‐out option is associated with two problems. First, it is likely to introduce blurring in encoding direction that can hamper the spatial specificity of identified perfusion changes (Liang et al., 2014). Second, it can lead to loss of signal in areas around the ventricles and thereby affect the representation of perfusion of surrounding structures (unpublished pilot study). The fact that we did not find any perfusion change in the ACC or thalamus might have been caused by the latter artifact. Both types of artifacts can be reduced by segmenting the read‐out (Alsop et al., 2014), however, usually at the cost of temporal resolution. Thus, in order to acquire a desired number of images, more time is needed, or in the case of measurement time constraints, fewer images can be collected and thereby relative SNR and power will decrease.

The relative SNR is also influenced by the number of sampling points, i.e. stimulus duration. The duration of our pain stimulation might have been too short to activate areas of the nociceptive network that we missed. Overall, ASL has a much lower SNR compared to BOLD fMRI. Specifically, only approximately 1% of the total signal is caused by blood delivered to the tissue (Liu & Brown, 2007). Both the lack of match between individual pain thresholds and stimulus intensity, and the relatively short stimulation time, limited the power of our experimental paradigm.

To conclude, using ASL we found that a single stimulation block of 96 seconds inducing sharp mechanical pain is sufficient to increase perfusion in many parts of the pain matrix (S1, S2, insula, temporal and parietal areas, and cerebellum). Moreover, with our paradigm, we found a positive correlation between pain intensity ratings and perfusion increase in the posterior insula during mechanical noxious stimulation. In future studies, longer stimulation duration, matching of subjective stimulus intensity level and modifications in the sequence read‐out options may result in enhanced power to detect activation within other pain regions (e.g., ACC, PFC, thalamus, amygdala). The specificity of acute sharp mechanical pain, with respect to regional changes in blood flow compared to other forms of acute pain, needs to be investigated in future studies. In the field of pain research in general and with regard to pain processing in BPD specifically, ASL constitutes an interesting alternative to BOLD fMRI, since it is well suited for paradigms with low task frequency.

## CONFLICT OF INTEREST

The authors declare no conflict of interest.

### PEER REVIEW

The peer review history for this article is available at https://publons.com/publon/10.1002/brb3.2442.

## Data Availability

The data are not publicly available due to privacy or ethical restrictions.
